# Effect of smoking and finishing and polishing protocol on color stability and surface roughness of resin composite

**DOI:** 10.1186/s12903-025-07161-1

**Published:** 2025-12-05

**Authors:** Mohamed Samy Salama, Sara Adel Botros, Fatma Makkeyah, Mohamed Shamel, Mahmoud Al Ankily

**Affiliations:** 1 Restorative Dentistry Department, Faculty of Dentistry, The British University in Egypt, Cairo, 11837 Egypt; 2 Fixed Prosthodontics Department, Faculty of Dentistry, The British University in Egypt, 11837 Cairo, Egypt; 3 Oral Biology Department, Faculty of Dentistry, The British University in Egypt, 11837 Cairo, Egypt

**Keywords:** Smoking, Cigarettes, Heated tobacco product, Finishing and polishing, Color stability, Surface roughness, Resin composite

## Abstract

**Purpose:**

To investigate the influence of smoking and finishing and polishing protocol on color stability and surface roughness of resin composite.

**Materials and methods:**

Seventy-two discs fabricated from supra-nanofilled composite were divided to 6 groups according to: Tobacco Product (Conventional Cigarette/CS and Heated Tobacco/HTP); and Finishing and Polishing Protocol (Control/C, Multi-step finishing system/FS, and Medium-grit abrasive bur + two-step polishing system/PS). After finishing and polishing, specimens were exposed to a total of 600 cigarettes, divided into 20 each day, simulating 30 days of smoking. Color parameters were detected using a spectrophotometer. Surface roughness (Ra) was measured before and after smoking exposure using a surface roughness tester. Surface morphology was assessed under SEM. Data were analyzed using ANOVA/Tukey’s and student t-test.

**Results:**

For CS, ∆E values of C group was significantly higher than both finishing/polishing groups, which were statistically similar. For HTP, no significant difference in ∆E values between all groups. ∆E values of CS were significantly higher than HTP in all groups. For CS, C group showed significantly higher change in Ra than FS group; whilst no significant difference in Ra change values of PS and those of C and FS groups. For HTP, no significant difference in Ra change between all groups. Within C group, Ra change of both smoking groups was statistically similar. Within FS and PS groups, change in Ra of HTP was significantly higher than CS.

**Conclusion:**

Conventional cigarette smoking resulted in pronounced color change in the supra-nanofilled composite. While heated tobacco product produced an increased surface roughness. Both multi-step finishing system and two-step polishing system displayed comparable color change and surface roughness in supra-nanofilled composite.

## Introduction

Tooth discoloration is one of the chief esthetic concerns as it influences the physical and social appeal of the patient. Particularly, smokers’ teeth tend to develop tobacco stains resulting in tooth discoloration and restoration staining [1]. Tooth discoloration is classified as intrinsic or extrinsic discoloration, where extrinsic staining are caused by external factors that adhere to the tooth surface, like tobacco stains [2]. The color change of restorations following smoking represent one of the common causes of restoration replacement [3, 4]. 

Cigarettes are considered the most commonly used form of tobacco. Tobacco stains are manifested as a black or dark brown discoloration covering the gingival third of teeth [5, 6]. Although, cigarette smoke carries several toxic substances [7], yet tar is the most important ingredient of tobacco that causes discoloration [8]. Thermal effects of cigarette smoke might be also another reason of discoloration [9]. Nowadays, recent nicotine products such as electronic cigarettes and heated tobacco products have become dramatically widespread. Yet, staining potential of such products and actual tobacco components causing discoloration is still unknown [10, 11]. 

For direct restorations, resin composite restorative materials have been widely recommended due to their esthetic appeal. Despite mechanical and esthetic improvements, color instability remains the main concern of resin composites. Color stability of resin composites could be affected by the size of fillers, resin matrix type, polymerization depth and coloring agents [12]. 

On the other side, surface roughness of resin-based restorative materials has been shown to be also influenced by size, distribution, and quantity of filler particles, and composition of resin matrix [13]. It has been reported that small-sized filler particles exhibited better polishability, and hence lower color change [14]. Meanwhile, the method of finishing and polishing used has a great impact on the surface quality, as improper finishing and polishing can result in excessive plaque accumulation and increased surface staining and degradation [15]. 

Hence, the purpose of this study was to investigate the influence of smoking of conventional cigarettes and recent heated tobacco products along with different finishing and polishing protocols on color stability and surface roughness of resin composite restoration. The null hypothesis suggested was that neither smoking nor finishing and polishing protocol would affect color stability and surface roughness of resin composite.

## Materials and methods

### Materials

All products’ description, composition, manufacturer and lot number are presented in Table 1.

**Table 1:** Products’ description, composition, manufacturer and lot number

Products	Description	Composition	Manufacturer	Lot Number
Palfique LX5	Supra nano-filled composite	-Filler: 82% wt, 71% vol. Silica-zirconia filler and composite filler. Particle size range: 0.1 to 0.3µm. -Resin: Bis-GMA and Triethylene glycol dimethacrylate.	Tokuyama Dental Corporation Inc., Japan	281E53
Diamond Master Discs	Multi-step diamond finishing and polishing discs	-Diamond sandpaper disks in coarse, medium, and fine grits. -Diamond Flex felt disks: micro bristles micronized diamond.	FGM, Joinville, Santa Catarina, Brazil	4000000266
Diacomp Plus Twist	Two-step polishing system	Flexible plastic lamellae with embedded diamond grains of ≈20 μm. -Pre-polishing medium polisher (Pink): 40-50 μm. -High-shine fine polisher (Gray): 3-6 μm.	EVE, Germany	486732
Diamond Excel polishing paste	Diamond polishing paste	Micronized diamond-based polishing paste with extra-fine grit (2-4 μm).	FGM, Joinville, Santa Catarina, Brazil	4000002091

## Methods

### Samples size calculation

The sample size was calculated using G*Power 3.1.9.2 Software. For ∆E, sample size was estimated using data obtained from a previous study [4]. Based on a mean difference of 4.0 ± 0.8, the calculated effect size was 2.94 using a power of 95% for unpaired t-test and two-tailed significance level of 5%. The estimated sample size was 4 composite specimens per group, which was increased by 30% to 6 composite specimens in each group (*N* = 36 per 6 groups). For surface roughness, sample size was calculated based on a previous study [16] reporting a 0.335 ± 0.035 μm mean difference. Based on an effect size of 2.91 with a 5% significance level and a 95% test power, a minimum of 5 composite specimens were required per group, which was increased by 20% to 6 composite specimens per group (*N* = 36 per 6 groups).

### Study design

This research proposal was approved by the Research and Ethics Committee of the Faculty of Dentistry, The British University in Egypt, with approval no. 24–057. Seventy-two discs fabricated from a supra-nanofilled composite were randomly divided to 6 groups (*n* = 12) according to study variables: (1) Tobacco Product (conventional cigarettes/CS; and heated tobacco product/HTP); and (2) Finishing/Polishing Protocol (control/C; multi-step finishing discs/FS; and Medium-grit abrasive bur + two-step polishing system). Half of the specimens of each study group (*n* = 6) was assigned for color stability assessment and the other half (*n* = 6) for surface roughness measurements.

### Specimens’ preparation

**Fig. 1:**
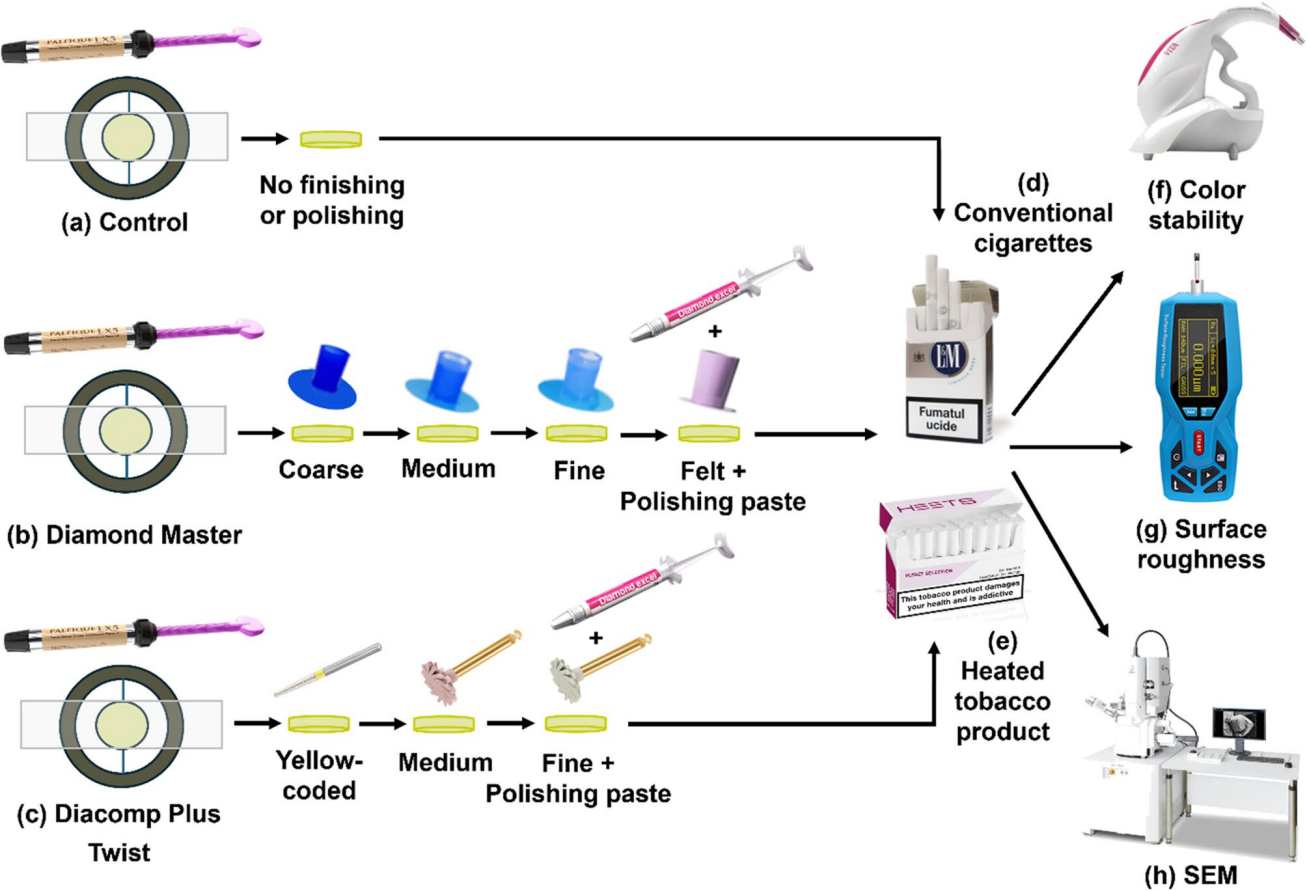
Diagrammatic illustration of specimen preparation procedures. Specimen preparation steps of (**a**) Control group, (**b**) Multi-step finishing system group (Diamond Master), and (**c**) Two-step polishing system group (Diacomp Plus Twist). Specimen exposure to (**d**) conventional cigarettes smoking (LM Blue) and (**e**) heated tobacco products (Heets Russet). Analysis of (**f**) color stability, (**g**) surface roughness and (**h**) surface morphology under SEM before and after smoking

A diagrammatic illustration of specimens’ preparation procedures is presented in (Fig. 1). Seventy-two composite discs (Palfique LX5, Tokuyama, Japan) of shade A2, 2 mm in thickness x 10 mm in diameter, were fabricated in a specially-designed split Teflon mold. A celluloid strip was fixed on a glass slab, over which the Teflon mold was placed. Composite was packed inside the mold and then another celluloid strip and glass slab were placed over the top surface of the composite with gentle finger pressure in order to allow excess material to escape and provide a smooth surface. Light polymerization was performed through the strip to prevent formation of oxygen inhibited layer for a total of 40 s, 20 s from top surface and 20 s from bottom surface, using a LED light-curing device (Elipar™ Deep Cure-L, 3 M ESPE, USA) at a light intensity of 1200 mW/cm^2^. Light intensity of the curing device was regularly checked with a radiometer (Litex 682, Dentamerica Industry, USA). The cured specimens were stored in distilled water for 24 h at 37 °C.

### Finishing and polishing procedures

A diagrammatic illustration of finishing and polishing protocols is presented in Fig. (1). The top surfaces of the composite discs were used as the experimental surfaces. To minimize variations, a single operator executed all finishing and polishing protocols with electric low speed contra-angled handpiece with transmission ratio 1:1 (T1 Line C 40 L, Dentsply Sirona, Germany). The specimens were held on split mold during finishing and polishing protocols. Pressure was maintained at 30–40 gm (≈ 0.3 N) using a kitchen scale [17]. For standardization, each finishing and polishing step was performed in 10 strokes for 20 s in one direction in a planar motion, in order to permit comparison among them [18]. 

#### Group 1: control group (C)

Composite discs were cured against celluloid strip (TOR-VM, Russia) without finishing or polishing.

#### Group 2: multi-step finishing system (FS)

Sandpaper discs in coarse, medium, and fine grits (Diamond Master, FGM, Brazil) were used in descending sequence, each for 20 s under dry condition. After each disc, specimens were rinsed with air-water spray for 10 s, then air-dried for 5 s. A diamond polishing paste (Diamond Excel, FGM, Brazil) was used with Diamond Flex felt disks as a final polishing step. Since there was no recommendation for rpm by the manufacturer, finishing was performed at 10,000 rpm as the rpm of most finishing disc systems range from 10,000 to 20,000 RPM [19]. Each finishing disc was discarded after single use.

#### Group 3: two-step polishing system (PS)

Prior to polishing, specimens were finished with super-fine diamond bur (Komet, Germany) with 25 μm particle grit size for 15 s under water coolant at 40,000 rpm as per the manufacturer recommendation. Specimens were subsequently polished using medium (pink) and fine (grey) polishing wheels (DIACOMP PLUS TWIST, EVE, Germany) sequentially at 10,000 rpm for 20 s each with the conjunction of the diamond polishing paste with no water cooling [19, 20]. After each step, specimens were rinsed with air-water spray for 10 s, then air-dried for 5 s. Each finishing burs and polishing wheel was discarded after 3 uses.

### Exposure to smoking

**Fig. 2:**
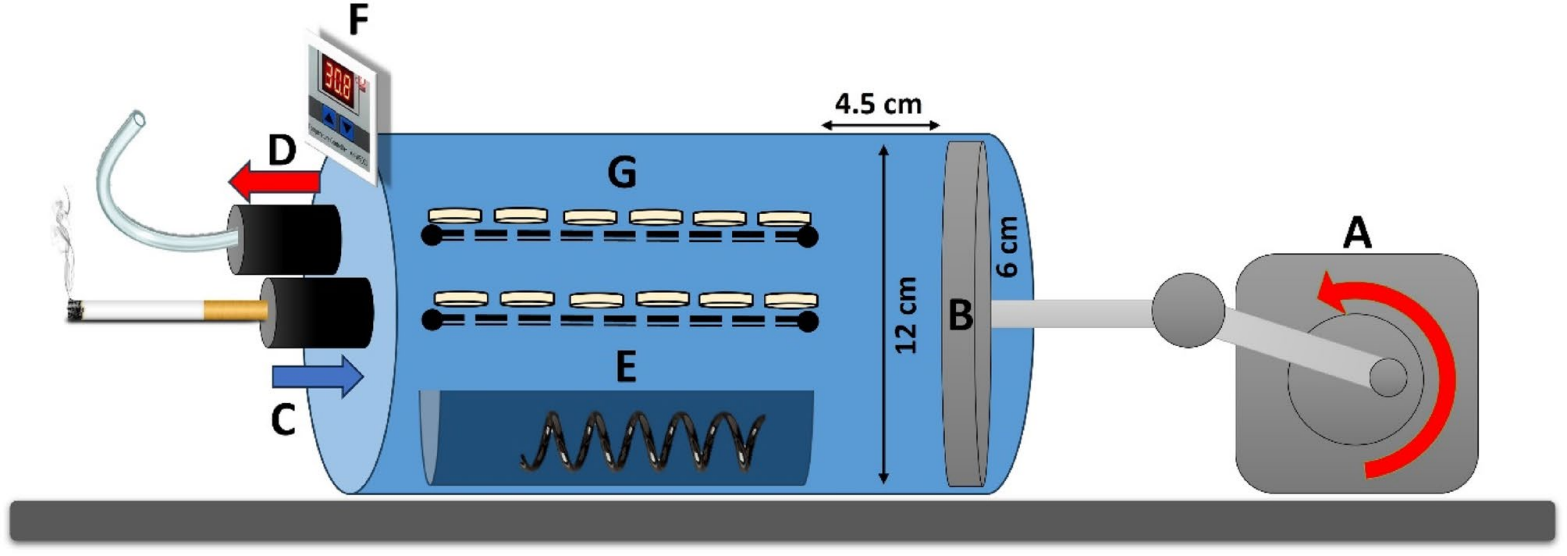
The smoking apparatus consists of (**A**) a gearbox to slow down the motor speed to 2 Hz (2 cycles/sec) with a crankshaft attached to a slider to change the movement to linear instead of rotation of 4.5 cm distance. **B** A stainless steel hollow chamber of 12 cm internal diameter with a piston to produce a suction power of 500 ml volume that simulates tidal volume taking during smoking. The cigarettes or electronic smoking device were attached to (**C**) an inhalation valve allowing unidirectional smoke inflow. **D **An exhalation valve which allows unidirectional smoke outflow. **E** A water container with a heater placed at the bottom of the device, which was connected to (**F**) a thermal sensor to adjust temperature at 37°C and 100% humidity similar to oral cavity. Specimens were placed on (**G**) perforated trays to allow penetration of smoke

Conventional cigarettes, CS (LM, Philip Morris International Inc., Egypt) and heated tobacco products, HTP (Heets, Russet, Philip Morris International Inc., Italy) were used. Specimens were placed in a specially designed smoking apparatus [10, 21–23] (Fig. 2) and exposed to 600 cigarettes/sticks, divided to 20 cigarettes each day, which represented 30 days of medium smoking behavior [24]. Afterwards, specimens were thoroughly rinsed with distilled water for 5 min in an ultrasonic cleaner (CD-4830, CODYSON, China) and dried with absorbent paper before color and surface roughness measurements.

### Color stability (ΔE)

Color measurements were evaluated before and after smoking exposure. Specimens were on a standard white background before color measurement procedures. Color properties were measured using a digital spectrophotometer (VITA Easyshade Advance 4.01, VITA shade, VITA, USA). The tip of the device was placed over the center of the specimen. Color parameters were recorded as L*, a*, and b* values; where L is the axis of lightness, a is the axis of chromaticity (green-red), and b is the axis of color (blue-yellow). The color change (ΔE) was calculated using the following formula: ΔE2 − 1 = ([ΔL]^2^ + [Δa]^2^ + [Δb]^2^)^1/2^. ΔE values are used to define color change in dental materials, and a ΔE value greater than 3.5 is considered clinically unacceptable [25]. 

### Surface roughness

Surface roughness was evaluated before and after smoking exposure. Surface roughness (Ra) was recorded using a profilometer (JITAI8101 Surface Roughness Tester - Beijing Jitai Tech Detection Device Co., Ltd., China). Three measurements were taken from each surface at 3 equidistant points from the center, and the overall mean value was calculated for each specimen. The change in surface roughness was determined by calculating the difference between initial and final measurements for each specimen.

### Surface morphology evaluation under scanning electron microscopy (SEM)

Surface morphology was evaluated using representative specimens from each group assigned for color change assessment before and after exposure to smoking. Specimens were vacuum-dried before examination. SEM examination was performed in Nanotechnology Research Center at The British University in Egypt using an environmental SEM (ThermoFisher, Quattro S Felid Emission Gun, Environmental SEM FEG ESEM, USA) in secondary electron mode at a standardized magnification x800.

### Statistical analysis

Statistical analysis was performed using IBM SPSS Statistics Software 25 for Windows (SPSS, Inc., Chicago, IL, USA). The significance level was set at *p* ≤ 0.05. Data was presented as mean and standard deviation (SD). Data normality was verified using Kolmogorov-Smirnov and Shapiro-Wilk test. Two-way ANOVA was used to investigate the effect of study variables on color stability and surface roughness. Intragroup comparisons were performed using One-Way ANOVA followed by post-hoc Tukey’s test. Intergroup comparisons were conducted using an independent student t-test.

## Results

### Color stability (ΔE)

One-Way ANOVA followed by post-hoc Tukey’s test (Table 2) displayed that there was a significant difference in ∆E values between finishing and polishing protocols within CS groups (*P* = 0.032). ∆E mean values of PS was significantly lower than control group. No significant difference was observed between control and FS groups, and between FS and PS groups. While within HTP groups, there was no significant difference between finishing/polishing protocols (*P* = 0.691). Independent student t-test (Table 2) showed that in all finishing/polishing protocols, CS displayed significantly higher ∆E mean values than HTP.

**Table 2:** Mean±St. Deviation for color change (∆E)

	Control	FS	PS	*P*-value
CS	11.93±2.65^a^	8.30±2.37^ab^	8.14±2.52^b^	0.032*
HTP	2.31±0.76	2.43±1.24	2.94±1.82	0.691NS
*P*-value	<0.001*	0.001*	0.003*	

Mean values with different superscript letters within the same row are significantly different

*Significant (*p≤*0.05); NS: non-significant (*p*>0.05)

### Surface roughness

One-Way ANOVA followed by post-hoc Tukey’s test (Table 3) showed that there was a significant difference in roughness mean change between finishing/polishing protocols within CS groups (*P* = 0.026). Ra mean change of control was significantly higher than FS. No significant difference was detected between control and PS, and between FS and PS. While for HTP, there was no significant difference between finishing/polishing protocols (*P* = 0.734). Within control group, no significant difference in Ra values between CS and HTP. Whereas in FS and PS groups, Ra values of CS were significantly lower than HTP.

**Table 3:** Mean±St. Deviation for mean change in surface roughness Ra (μm)

	Control	FS	PS	*P*-value
CS	0.09±0.18^a^	0.02±0.01^b^	0.06±0.07^ab^	0.026*
HTP	0.08±0.05	0.10±0.07	0.11±0.08	0.734NS
*P*-value	0.880NS	0.018*	0.012*	

Mean values with different superscript letters within the same row are significantly different

*Significant (*p≤*0.05); NS: non-significant (*p*>0.05)

### Surface morphology evaluation

**Fig. 3:**
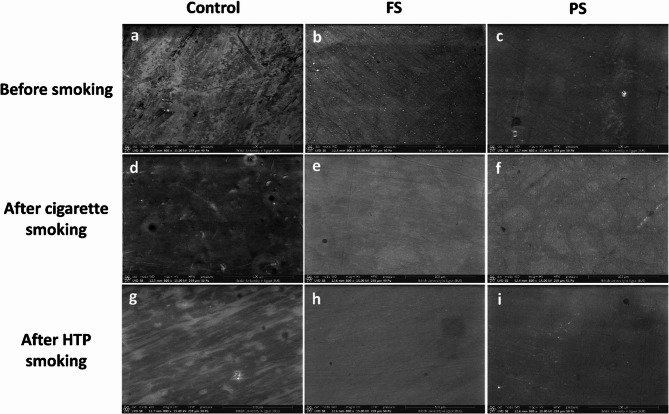
SEM photomicrographs of composite top surfaces at magnification X800 of different finishing and polishing groups (C, FS, PS) before and after exposure to smoke

The surface morphology micrographs of the studied specimens at 800X are presented in (Fig. 3). Prior to smoking exposure, control group showed a rougher surface compared to both finishing and polishing groups which produced a more homogenous surface with the formation of a resin smear layer along with the presence of some multidirectional scratches.

After exposure to cigarette smoking, both finishing and polishing groups displayed smoother composite surfaces when compared to control group which showed an irregular surface topography. Deposition of organic materials on the surface was evident in all groups.

After exposure to HTP smoking, control group showed comparable surface changes with both finishing and polishing groups.

## Discussion

Color change of resin composite materials could be generally caused by the following reasons: (1) extrinsic factors such as plaque accumulation, smoking and dietary habits; (2) [26] surface alterations related to type of finishing/polishing system promoting surface degradation and adsorption of coloring agents; [27] and (3) intrinsic factors related to chemical composition and alterations in matrix/filler interphase of the material [26, 28]. Since dental esthetics is of great concern nowadays, and since one of the crucial reasons for composite restoration replacement is staining and color change, the purpose of the present study was to assess color stability and surface roughness of a supra-nanofilled resin composite submitted to different smoking products (with 0.5 mg nicotine) and finishing/polishing protocols. The supra-nanofilled resin composite was selected due to its established capability to produce a smoother surface finish relative to nanofilled and microhybrid composites, as indicated in a previous research by Qurany et al. (2024) [29]. This enabled a more precise assessment of the primary variable, which is the influence of conventional and heated tobacco products on the composite physical properties.

In literature, there are few studies evaluating the effect of heated tobacco products on resin-based restorations [23, 30]. In addition, not many studies have standardized the method of exposing restorative materials to cigarette smoke, such as the equipment design, number of cigarettes, smoke flow mechanism, smoke exposure time, simulation of oral cavity temperature and humidity [4, 31–33]. In contrast to these studies, the design methodology of the current study was to use a specially designed apparatus with a suction power capacity of 500 mL volume similar to tidal volume per each respiratory cycle during smoking. The apparatus received one cigarette at a time permitting simultaneous unidirectional smoke inhalation and exhalation at a standardized time interval and speed. In order to simulate the oral cavity, temperature was set to 37 °C at 100% humidity by the aid of a thermal regulator.

To maintain methodological consistency with the existing literature, especially studies assessing the impact of smoking and finishing/polishing on dental composites, CIELab (ΔE*ab) formula was chosen over ΔE00 to calculate color change (ΔE). This approach allowed for direct comparison with previous findings and facilitated a meaningful contribution to the ongoing studies on this topic, as also highlighted by Paolone, et al. (2022) [10]. While we acknowledge that ΔE₀₀ [34] is considered a more perceptually uniform formula in recent color research, studies adopting it typically differ in aim, scope, and methodological design from ours. Therefore, applying ΔE₀₀ in our study would not only limit the comparability with established findings but may also misrepresent our intended analysis framework.

Regarding color stability findings, a significant change in color (∆E = 8.14–11.93) was found in resin composite specimens exposed to conventional cigarettes, regardless of the finishing and polishing system, which was visually perceptible and considered clinically unacceptable [25]. On the contrary, all specimens exposed to heated tobacco products displayed a minor color change (∆E = 2.31–2.94), which was considered clinically acceptable [25]. Thus, the null hypothesis that smoking would have no effect on color stability of resin composite was partially rejected.

Cigarette smoke is made up of a mixture of particulate and volatile components [7]. The particulate phase mainly consists of tar, while the volatile phase includes carbon monoxide, carbon dioxide, nitrogen monoxide and water. Tar represents more that 90% of smoke products. The tar precipitates on cigarette filters turning their color into yellow-brown, could be more likely the main agent causing discoloration of teeth and restorations [3, 32]. Upon combustion of those elements, dark pigments and metals like cadmium, arsenic, nickel and lead are released [35]. Deposits of these elements may have adhered to top surfaces of composite specimens, resulting in staining and surface alterations [36]. More darkening and yellowing of the specimens were observed for conventional cigarettes group compared to HTP group, interpreted by the significant reduction in L∗ coordinate and increase in a∗ and b∗ coordinates. This agrees with a previous study showing a prominent discoloration effect of conventional cigarette smoke on artificial denture teeth in comparison to HTP smoke.

The reduced staining potential of HTP could be attributed to the higher aerosols generated from heated sticks when compared to cigarette smoke [37]. HTP device used in the current study consisted of a tobacco heating system different from the burning process that occurs with a lighted cigarette, thus producing less harmful ingredients. The combustion of conventional cigarettes may reach a temperature higher than 600 °C [38], whereas HTP can be heated up to 350 °C [39]. When heated instead of burning, aerosols of suspended liquid droplets are liberated, thus eliminating the formation of any carbon-based solid particulates [40]. These aerosols showed a different chemical composition, composed primarily of water, nicotine and glycerin [41]. Nicotine is colorless in its original form, however, its oxidized state can cause yellowish staining [1]. This might explain the reduced discoloration effect on specimens exposed to HTP smoke in comparison to conventional cigarette smoke, irrespective of the finishing/polishing protocol.

Within conventional cigarettes groups, control specimens showed higher color change compared to both finishing and polishing groups. Meanwhile, there was no significant difference in ∆E values between control and finishing and polishing groups in heated tobacco products groups. Hence, the null hypothesis that finishing and polishing protocol would not affect color stability of resin composite was partially accepted. The fact that control specimens cured against polyester strip presented more staining than those submitted to either finishing and polishing protocol may be justified by the superior homogeneity of the composite surface resulting after finishing and polishing, rendering the surface more regular [42, 43]. As proved in a previous study [44], smoother composite surfaces produced from the adaptation of polyester strip are not necessarily more resistant to discoloration. In contrast, photo-polymerization of control specimens under pressure of polyester strip, may have created a superficial layer rich in organic matrix with lower filler loading [45]. Thus, this unpolished surface became unstable and have readily adsorbed the pigments emitted from the combustion products of cigarettes smoke. These findings came in accordance with those of a previous study which demonstrated an increased potential of cigarette smoke to stain all composite samples in the absence of polishing, along with increased surface roughness [35]. 

Regarding surface roughness, both smoking groups showed statistically similar mean change in Ra in control specimens. Whereas, specimens exposed to heated tobacco products showed significantly higher Ra mean change compared to conventional cigarettes in both finishing and polishing groups. Therefore, the null hypothesis that smoking would not affect composite surface roughness was partially rejected. As previously mentioned, the absence of finishing and polishing renders the composite surface more irregular and rougher owing to uneven matrix-filler distribution [42, 43]. Since heated tobacco system heats tobacco without burning/combustion to release nicotine-containing aerosols, the composition of the aerosol is greatly different form the smoke liberated by combustible products of conventional cigarettes [37, 38]. IQOS aerosol primarily contains water which comprises more than 80% [39]. Due to this high water content, aerosols formed from the condensation of water vapor caused wetting of composite surfaces. The hydrophilic character of TEGDMA monomer present in the composition of the tested resin composite is expected to increase water sorption [46]. The additional presence of Bis-GMA monomer could also form, through its OH-groups, hydrogen bonds to water adsorbed on the surface [46]. Water acts as a polymer plasticizer, leading to hydrolytic degradation and surface deterioration of resin composite. Hence, adsorbed water could have weakened intermolecular bonds of resin matrix causing swelling of polymer network; minimizing frictional forces between polymer chains [47]. Moreover, a number of organic acids were identified in the total particulate matter of IQOS mainstream. The acidic pH of IQOS smoke originating from the combination of nicotine salts with organic acids [48], resulting in protonation and release of H + ions which may have broken C-C bonds of polymer matrix as well as siloxane bonds connecting matrix to fillers; thereby releasing filler particles leading to exposure of resin matrix [49]. Besides thermal effects of emitted aerosols, this could have assumably contributed to increasing surface roughness values of all specimens in HTP groups [4]. 

As for the conventional cigarettes groups, mean change in Ra values in control group was higher than two-step polishing system, followed by multi-step finishing system. On the contrary, no significant difference in Ra mean change between control and finishing and polishing groups when exposed to heated tobacco products. Hence, the null hypothesis that finishing and polishing protocol would have no influence on surface roughness was partially rejected. Our findings were in agreement with the study of Itanto et al., [50] who reported an improved surface finish of a nanofilled composite resin after using a multi-step finishing system. This could be attributed to the flexibility, composition and technique of usage of the aluminum oxide discs [50]. The malleability of such discs promotes better adaptation and homogenous abrasion of both filler particles and resin matrix, preventing filler displacement from the matrix [50]. This system is also structured to be used in a sequential order of descending abrasiveness level, starting with coarse, medium, fine grains, and finalizing with felt discs in a unidirectional planar movement; thereby promoting a smoother final surface texture. In the two-step polishing protocol, a pre-roughening step with diamond burs was performed, to simulate the clinical situation. The fine-grit yellow-coded abrasive burs equivalent to the coarse aluminum discs of the multi-step system were selected, in order to minimize scattering of the initial roughness values between both groups. This finishing step was followed by pre-polishing with medium-grain spiral, then final polishing with fine-grain spiral.

Discoid shape polishers were used in the current study aiming to exclude the effect of tool geometry on surface roughness results. This was essential for standardizing specimen geometry and minimizing experimental variability. In order to obtain accurate surface roughness readings using profilometer, specimens should have flat surfaces [51]. Hence, the specimens fabricated in this study were flat discs, which might be relatively easier for the wheel-shaped polishing tips to come in direct contact with the flat surface of the specimens, producing a high shine finish [42]. It was also concluded in a former study that diamond particles impregnated within the flexible rubber material of the polishers have higher toughness and hardness than aluminium oxide and silicone carbide particles, which may have attributed to more homogenous abrasion and effective polishing [52, 53]. These results came in accordance with those of Di Silva et al., [16] who claimed that EVE Diacomp Twist Plus was effective in reducing surface roughness. However, theses findings did not follow those of Elgammal et al., [54] who reported high surface roughness results with the two-step polishing system. This disparity could be related to the difference in the type of composite material tested, as they were using a bulk fill resin composite.

To ensure standardization of finishing and polishing procedures, all steps were executed by the same operator following respective manufacturers’ recommendations regarding speed and motion. Moreover, the applied pressure during polishing procedures was controlled at light pressure of approximately 30–40 g using a kitchen scale. A diamond polishing paste was used with final polishing step of both systems as several studies [55] proved its efficacy in providing a smoother and glossier surface finish. This is largely due to the great wear capacity of the diamond paste as diamond particles are harder than composite filler particles [55, 56]. Consequently, this would enable equal and uniform removal of both composite phases, hence, minimizing surface roughness. This was supported by SEM photomicrographs of conventional cigarettes group, which illustrated smoother surfaces of specimens subjected to both finishing and polishing systems when compared to control group. The F/P systems appeared to produce a resin smear layer on the composite surfaces with some multidirectional scratches. In the present study, all surface roughness values, irrespective to smoking or finishing and polishing protocol, were lower than 0.2 μm, which is the critical size for bacterial adhesion. These values were in accordance with the surface quality standard ISO 1302:2002 [57]. 

Limitations of the present study include lack of assessment of different brands and flavors, varying nicotine concentrations, and tooth brushing simulation during smoke exposure. Future studies are also recommended to investigate the effect of other commercially available finishing and polishing systems and polishing pastes with different particle sizes on color stability and surface topography of resin composite. Additionally, further studies could include other composite types with varying filler size/content and resin matrix composition in order to validate the broader applicability of the current findings. Reporting color change data using ΔE00 formula across a broader spectrum of materials should be considered in future research. Furthermore, it is important to consider the interaction of aging simulations with other factors, such as dietary habits and oral hygiene practices, to provide a more comprehensive understanding of their combined impact on resin composite materials.

## Conclusion

Under the limitations of the current study, it could be withdrawn that:

1. Conventional cigarette smoking resulted in a pronounced color change in supra-nanofilled composite compared to heated tobacco product.
2. Smoking of heated tobacco product produced an increased surface roughness compared to conventional cigarettes.
3. Both multi-step finishing system and two-step polishing system showed comparable color change and surface roughness in supra-nanofilled composite.

## Data Availability

The data supporting the findings of the present study are available within the article.
